# Supplementary material for: The alarmin IL33 orchestrates type 2 immune-mediated control of thymus regeneration
Source: Nat Commun. 2023 Nov 8;14:7201. doi: 10.1038/s41467-023-43072-x (PMC10632327; doi:10.1038/s41467-023-43072-x)
Supplement: Supplementary file 1 — Supplementary Information [file 41467_2023_43072_MOESM1_ESM.pdf]

# Supplementary Materials for

# The Alarmin IL33 Orchestrates Type 2 Immune-Mediated Control of Thymus Regeneration

Emilie J. Cosway[1], Kieran D. James[1], Andrea J. White[1], Sonia M. Parnell[1], Andrea Bacon[1], Andrew N. J. McKenzie[2], W. E. Jenkinson[1], and Graham Anderson[1*]

**Affiliations:**

[1]Institute of Immunology and Immunotherapy, University of Birmingham, Birmingham, UK.

[2]MRC Laboratory of Molecular Biology, Cambridge, UK

*Correspondence to g.anderson@bham.ac.uk

**This PDF file includes:**

Figs. S1, S2, S3, S4, S5, S6, S7, S8, S9, S10, S11, S12

**a**

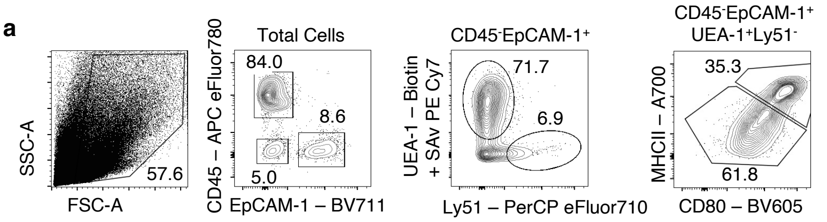


**Figure S1. Gating strategy for thymic epithelial stroma populations in thymus.** Populations were identified as follows: cTEC ($CD45^-EpCAM\text{-}1^+UEA\text{-}1^-Ly51^+$), mTEC ($CD45^-EpCAM\text{-}1^+UEA\text{-}1^+Ly51^-$), $mTEC^{hi}$ ($CD45^-EpCAM\text{-}1^+UEA\text{-}1^+Ly51^-MHCII^+CD80^+$), $mTEC^{lo}$ ($CD45^-EpCAM\text{-}1^+UEA\text{-}1^+Ly51^-MHCII^-CD80^-$. This gating strategy was used for Fig 1a, 1b, 2b, 2c, 3b, 3c, 5b and 7k.

**a**

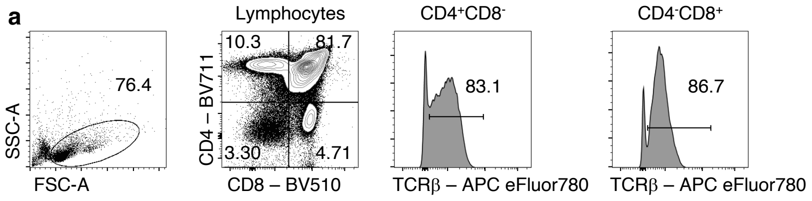


**Figure S2. Gating strategy for thymocytes.**

Populations were identified as follows: $CD4^{+}TCR\beta^{hi}$ ($CD4^{+}CD8^{-}TCR\beta^{hi}$), $CD8^{+}TCR\beta^{hi}$ ($CD4^{-}CD8^{+}TCR\beta^{hi}$) and DP ($CD4^{+}CD8^{+}$). This gating strategy was used for Fig 1a, 1b, 2c, 3c and 7k.

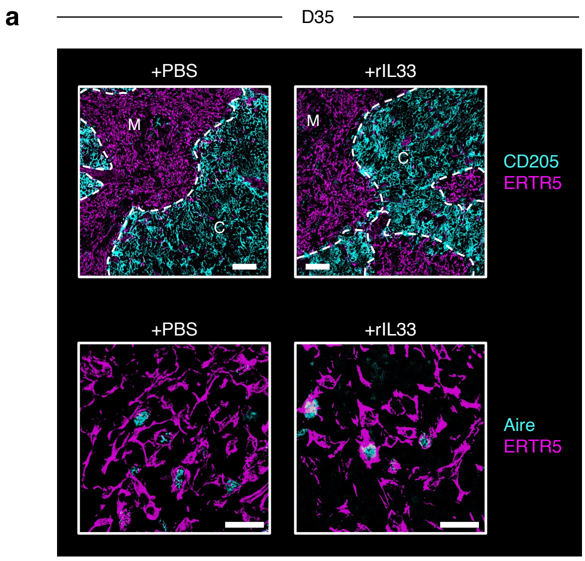


**Figure S3. Organisation Of Thymus Microenvironments Post-SLI Following rIL33 Addition.** (A) Balb/c mice were sublethally irradiated and injected with either PBS or rIL33 as show in Figure 2. Mice were harvested 35 days after SLI, and thymus sections were analysed by confocal microscopy for thymus organisation using antibodies to mTEC (ERTR5), cTEC (CD205) and Aire. Data is representative of n=4 obtained from 2 independent experiments. Upper panel scale bar: o100μm lower panel scale bar: 20μm.

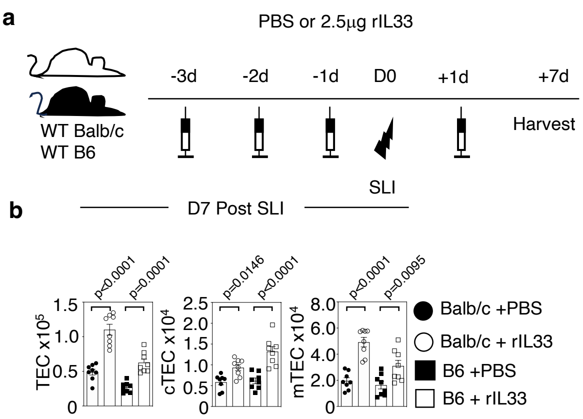


**Figure S4. Comparison of Effects of IL33 On Thymus Recovery In Balb/c and B6 mice After SLI.**

(A) Injection and SLI regime of mice for recovery analysis. (B) Quantitation of TEC, cTEC and mTEC populations of indicated mice with either PBE or IL33. All error bars show mean ± SEM, from an ANOVA using Šídák's multiple comparisons to compare PBS vs. rIL33 treated Balb/c mice or B6 mice. n=8 mice for each condition obtained from 2 independent experiments.

**a**

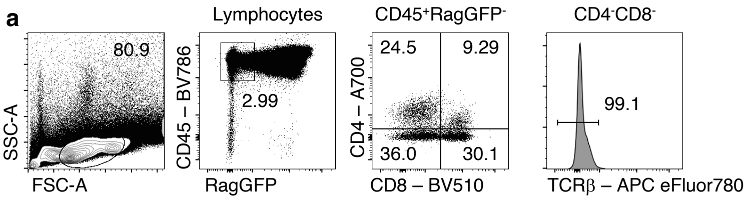


**Figure S5. Gating strategy for ILC.**
ILC were identified as $CD45^{+}RagGFP^{-}CD4^{-}CD8^{-}TCR\beta^{-}$. This pre-gating strategy was used to identify ILCs which were then further subdivided into ILC subsets.

a

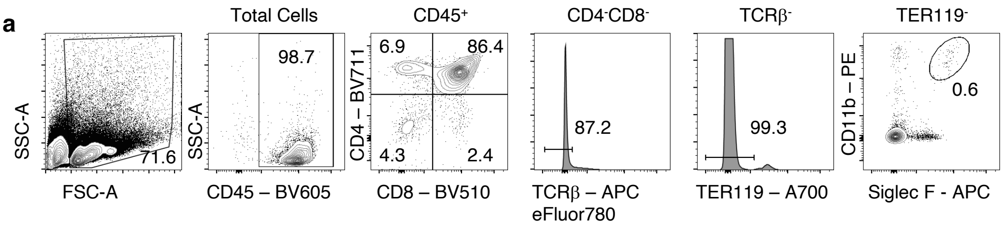


**Figure S6. Gating strategy for eosinophils in thymus.**

Eosinophils were identified as CD45$^+$CD4$^-$CD8$^-$TCRβ$^-$CD11b$^+$SiglecF$^+$. This gating strategy was used for Fig5c.

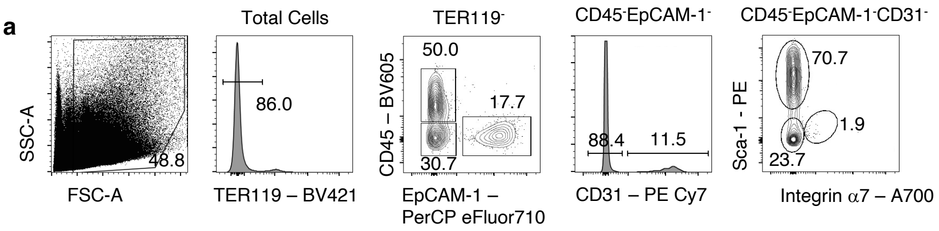


**Figure S7. Gating strategy for non-epithelial stroma populations in thymus.**
Populations were identified as follows: Endothelium (TER119$^-$CD45$^-$EpCAM-1$^-$CD31$^+$), Mesenchyme (TER119$^-$CD45$^-$EpCAM-1$^-$CD31$^-$Integrin$\alpha$7$^-$Sca-1$^{+/-)}$, Pericytes (TER119$^-$CD45$^-$EpCAM-1$^-$CD31$^-$Integrin$\alpha$7$^+$). This gating strategy was used for Fig 6b, 6c, 6d, 6f, 6g, 6i, 7g and 7i.

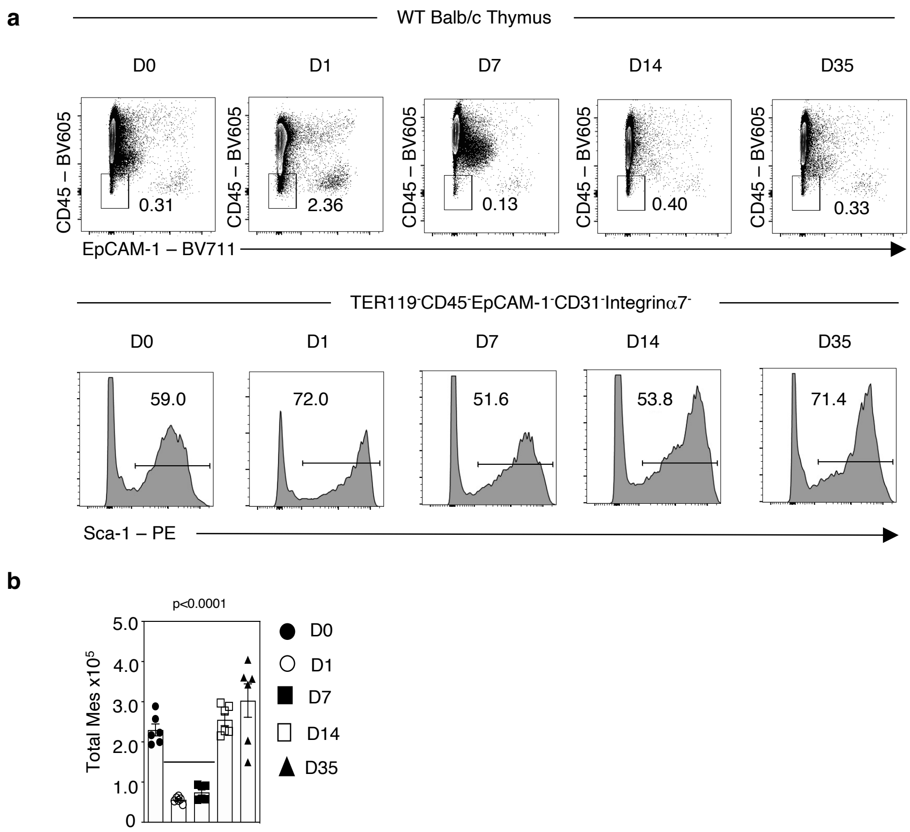


**Figure S8. Timeline of Mesenchymal Recovery Post SLI.**
(A) Representative FACs plots of total thymic mesenchyme, defined asCD45$^-$EpCAM-1$^-$TER119$^-$ CD45$^-$EpCAM-1$^-$CD31$^-$Integrin$\alpha$7$^-$, for Sca-1 expression at various points post SLI. (B) Analysis of total thymus mesenchyme in a similar timecourse after SLI, where statistics show mean ± SEM, comparing all timepoints to D0 from an ANOVA test with Dunnett's multiple comparisons test. Statistically significant data occurs at both D1 and D7. All analysis is from 6 mice for each time point, obtained from 2 independent experiments.

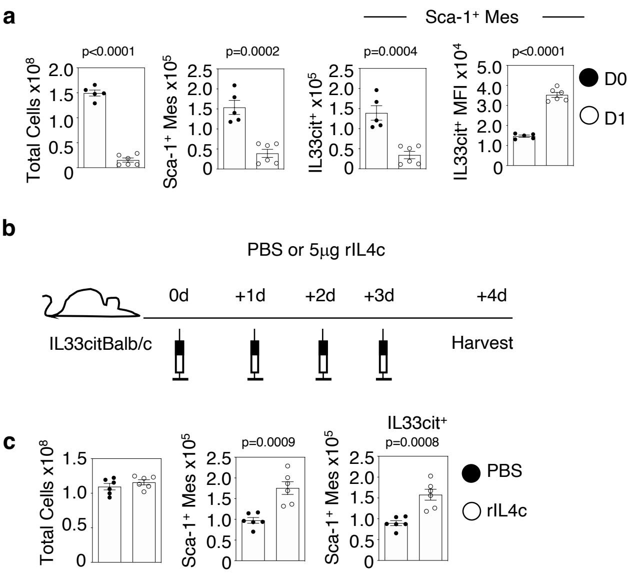


**Figure S9. Sca-1⁺ Mesenchyme Increase Expression of IL33cit After SLI and are Increased By rIL4c In The Steady State.**

(A) Quantitation of IL33cit expressing Sca-1⁺ mesenchyme D0 (n=5 animals) and D1 (n=6 animals) post SLI in IL33citBalb/c mice obtained from 2 independent experiments. (B) Injection regime of IL33citBalb/c mice with PBS or 5μg recombinant IL4-complexes, with analysis 1 day post final injection. (C) Analysis of the expression of IL33cit in IL33citBalb/c mice following rIL4-complex injections as in (B), with quantitation shown (C), n=6 animals for each condition obtained from 2 independent experiments.

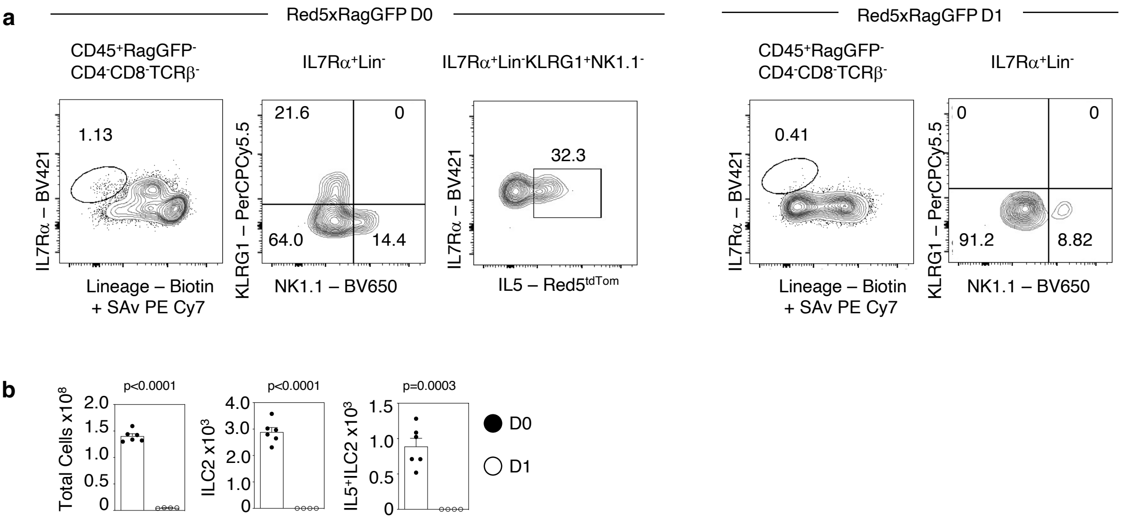


**Figure S10. ILC2 Are Depleted From The Thymus D1 After SLI.**
(A) Representative FACs plots for identification of ILC2 in Red5xRagGFP mice in the steady state (D0) and after SLI D1. (B) Quantitation of total thymus cells, ILC2 and IL5+ILC2. D0 n=6 and D1 n=4 animals obtained from 2 independent experiments. All bars show mean ± SEM from an unpaired two-tailed students t-test.

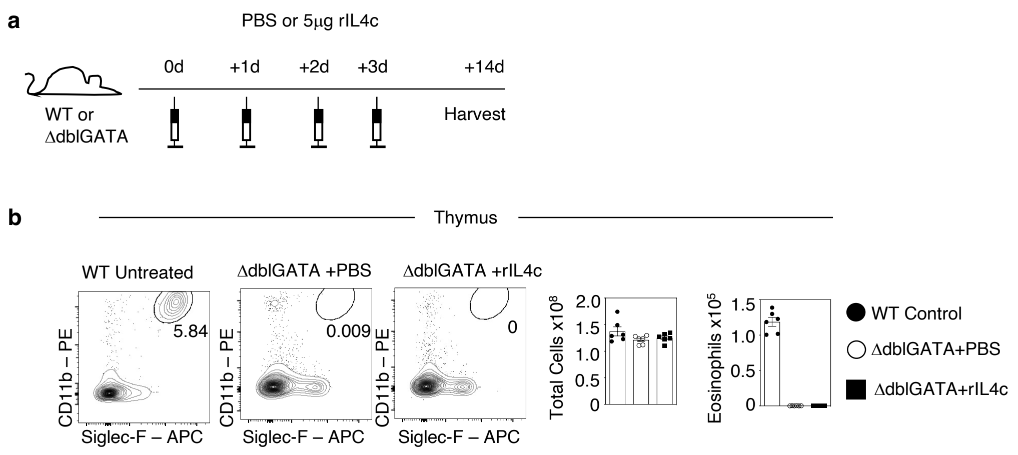


**Figure S11. Recombinant IL4c Does Not Rescue Eosinophil Development in ΔdblGATA Mice.** (A) Injection regime of WT and ΔdblGATA mice injected with PBS or rIL4c to assess induction of eosinophil development. (B) Representative FACs plots to show eosinophil populations in thymus of untreated WT Balb/c mice alongside ΔdblGATA mice injected with either PBS or recombinant IL4. Quantitation of eosinophils present in these different conditions is shown alongside total thymus cells. n=6 mice for each condition, obtained from 2 independent experiments.

a

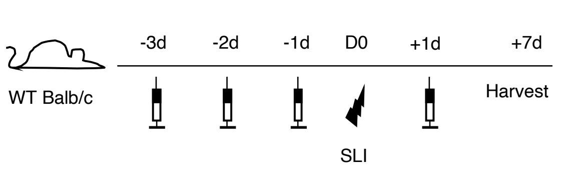


b

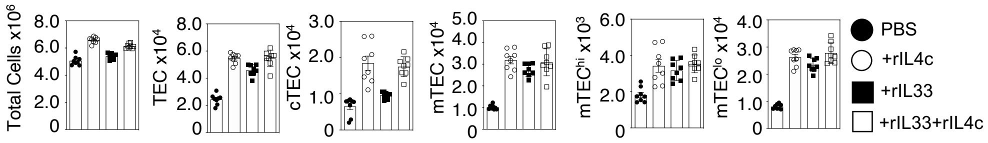


c

| ANOVA test with Šídák's multiple comparisons test | Total Cells | Total TEC | cTEC | mTEC | $mTEC^{hi}$ | $mTEC^{lo}$ |
|---|---|---|---|---|---|---|
| PBS vs. rIL4 | p<0.0001 | p<0.0001 | p<0.0001 | p<0.0001 | p=0.0002 | p<0.0001 |
| PBS vs. rIL33 | ns | p<0.0001 | ns | p<0.0001 | p=0.0015 | p<0.0001 |
| PBS vs. rIL33+rIL4 | p<0.0001 | p<0.0001 | p<0.0001 | p<0.0001 | p<0.0001 | p<0.0001 |
| rIL4 vs. rIL33 | p<0.0001 | p=0.0033 | p<0.0001 | ns | ns | ns |
| rIL4 vs. rIL33+rIL4 | p=0.0062 | ns | ns | ns | ns | ns |
| rIL33 vs. rIL33+rIL4 | p<0.0001 | p=0.0039 | p=0.0004 | ns | ns | p=0.0198 |

**Figure S12. Comparison of the Effects of IL4 and IL33, Either Individually Or in Combination, On Thymus Regeneration Post-SLI.** (A) Injection regime of mice with PBS/rIL33/rIL4c/rIL33+rIL4c for thymus recovery analysis. (B) Quantitation of total cells, TEC and TEC subsets following the indicated treatments. (C) Table of statistical analysis results of data in (B) whereby statistics show mean ± SEM, from an ANOVA test with Šídák's multiple comparisons test. n=8 mice for each condition obtained from 2 independent experiments.
