# Supplementary material for: The alarmin IL33 orchestrates type 2 immune-mediated control of thymus regeneration
Source: Nat Commun. 2023 Nov 8;14:7201. doi: 10.1038/s41467-023-43072-x (PMC10632327; doi:10.1038/s41467-023-43072-x)
Supplement: Supplementary file 3 — Reporting Summary [file 41467_2023_43072_MOESM3_ESM.pdf]

nature portfolio

Corresponding author(s): Graham Anderson

Last updated by author(s): 2023/10/20

Reporting Summary

Nature Portfolio wishes to improve the reproducibility of the work that we publish. This form provides structure for consistency and transparency in reporting. For further information on Nature Portfolio policies, see our Editorial Policies and the Editorial Policy Checklist.

Statistics

For all statistical analyses, confirm that the following items are present in the figure legend, table legend, main text, or Methods section.

n/a	Confirmed	
☐	☒	The exact sample size (*n*) for each experimental group/condition, given as a discrete number and unit of measurement
☐	☒	A statement on whether measurements were taken from distinct samples or whether the same sample was measured repeatedly
☐	☒	The statistical test(s) used AND whether they are one- or two-sided<br>*Only common tests should be described solely by name; describe more complex techniques in the Methods section.*
☐	☒	A description of all covariates tested
☒	☐	A description of any assumptions or corrections, such as tests of normality and adjustment for multiple comparisons
☐	☒	A full description of the statistical parameters including central tendency (e.g. means) or other basic estimates (e.g. regression coefficient) AND variation (e.g. standard deviation) or associated estimates of uncertainty (e.g. confidence intervals)
☐	☒	For null hypothesis testing, the test statistic (e.g. *F*, *t*, *r*) with confidence intervals, effect sizes, degrees of freedom and *P* value noted<br>*Give P values as exact values whenever suitable.*
☒	☐	For Bayesian analysis, information on the choice of priors and Markov chain Monte Carlo settings
☒	☐	For hierarchical and complex designs, identification of the appropriate level for tests and full reporting of outcomes
☒	☐	Estimates of effect sizes (e.g. Cohen's *d*, Pearson's *r*), indicating how they were calculated

Our web collection on statistics for biologists contains articles on many of the points above.

Software and code

Policy information about availability of computer code

Data collection	Flow cytometry data was acquired on a BD Fortessa Cell Analyser with FACs DIVA (v9.0) software. Confocal microscopy data was collected on a Zeiss LSM 880 microscope with Zen Black (14.0.22.201) software.
Data analysis	Flow cytometry data was analysed using FlowJo Version 10.8.1 and Graph Prism (v10.0.3). Confocal images were analysed using Zen Black (16.0.2.306).

For manuscripts utilizing custom algorithms or software that are central to the research but not yet described in published literature, software must be made available to editors and reviewers. We strongly encourage code deposition in a community repository (e.g. GitHub). See the Nature Portfolio guidelines for submitting code & software for further information.

Data

Policy information about availability of data

All manuscripts must include a data availability statement. This statement should provide the following information, where applicable:

- Accession codes, unique identifiers, or web links for publicly available datasets
- A description of any restrictions on data availability
- For clinical datasets or third party data, please ensure that the statement adheres to our policy

Authors confirm the data that supports the findings of this study are available in the figures and supplementary figures. Data generated in this study are provided in the accompanying Source Data file.

Research involving human participants, their data, or biological material

Policy information about studies with human participants or human data. See also policy information about sex, gender (identity/presentation), and sexual orientation and race, ethnicity and racism.

Reporting on sex and gender	N/A
Reporting on race, ethnicity, or other socially relevant groupings	N/A
Population characteristics	N/A
Recruitment	N/A
Ethics oversight	N/A

Note that full information on the approval of the study protocol must also be provided in the manuscript.

Field-specific reporting

Please select the one below that is the best fit for your research. If you are not sure, read the appropriate sections before making your selection.

- [x] Life sciences
- [ ] Behavioural & social sciences
- [ ] Ecological, evolutionary & environmental sciences

For a reference copy of the document with all sections, see nature.com/documents/nr-reporting-summary-flat.pdf

Life sciences study design

All studies must disclose on these points even when the disclosure is negative.

Sample size	Sample sizes are shown in the legends. They are based on previous similar published experiments on thymus regeneration (Cosway et al Science Immunology 2022) that were sufficient to reproducibly detect specific effects. Statistical methods were not used to predetermine sample size.
Data exclusions	No data was excluded.
Replication	Experiments were repeated, data is from at least two independent experiments giving similar results. Legends contain information on of numbers of experiments performed.
Randomization	Randomization was not performed, and to control for covariates we used litter mates where possible, and age- and gender-matched mice.
Blinding	Researchers were not blinded to mouse genotypes. This was because information on mouse genotype was required to allow appropriate grouping of control and experimental mice, and grouping of WT and genetically altered mice. This approach also ensured appropriate sample sizes were achieved by sacrificing the minimum number of mice required to achieve consistent data.

Reporting for specific materials, systems and methods

We require information from authors about some types of materials, experimental systems and methods used in many studies. Here, indicate whether each material, system or method listed is relevant to your study. If you are not sure if a list item applies to your research, read the appropriate section before selecting a response.

Materials & experimental systems

n/a	Involved in the study	
[ ]	[x]	Antibodies
[x]	[ ]	Eukaryotic cell lines
[x]	[ ]	Palaeontology and archaeology
[ ]	[x]	Animals and other organisms
[x]	[ ]	Clinical data
[x]	[ ]	Dual use research of concern
[x]	[ ]	Plants

Methods

n/a	Involved in the study	
[x]	[ ]	ChIP-seq
[ ]	[x]	Flow cytometry
[x]	[ ]	MRI-based neuroimaging

Antibodies

Antibodies used	anti-CD45, Brilliant Violet 786, 30-F11, mouse, eBioscience, 417-0451-82<br>anti-CD45, APC-eFluor 780, 30-F11, mouse, eBioscience, 47-0451-82

anti-CD45, Brilliant Violet 605, 30-F11, mouse, eBioscience, 406-0451-82
anti-TCRβ, APC-eFluor 780, H57-597, mouse, eBioscience, 47-5961-82
anti-CD11b, PE, M1/70, mouse, eBioscience, 12-0112-82
anti–Siglec-F, APC, ES22-10D8, mouse, Miltenyi Biotech, 130-123-816
anti–Siglec-F, Biotin, ES22-10D8, mouse, Miltenyi Biotech, 130-119-136
anti-CD4, Brilliant Violet 711, RM4-5, mouse, BioLegend, 100549
anti-CD4, FITC, RM4-5, mouse, BioLegend, 100509
anti-CD4, Alexa Fluor 700, RM4-5, mouse, BioLegend, 100536
anti-CD8α, Brilliant Violet 510, 53-6.7, mouse, BioLegend, 100751
anti-TER119, Brilliant Violet 421, TER-119, mouse, BioLegend, 116233
anti-TER119, Alexa Fluor 700, TER-119, mouse, BioLegend, 116220
mCD1d tetramer, APC, PBS-57, mouse, National Institutes of Health Tetramer Core Facility
anti-IL4RPE, I015F8, mouse, Biolegend, 144803
anti-IL7Rα, Brilliant Violet 421, A7R34, mouse, Biolegend, 135024
anti-IL7Rα, APC, A7R34, mouse, Biolegend, 135011
anti-KLRG1, PerCP/Cyanine5.5, 2F1/KLRG1, mouse, BioLegend, 138417
anti-NK1.1, Brilliant Violet 650, PK136, mouse, eBioscience, 416-5941-82
anti-CD3ε, PE/Cyanine 7, 145-2C11, mouse, Biolegend, 100319
anti-CD5, PE/Cyanine 7, 53-7.3, mouse, eBioscience, 25-0051-81
anti-CD11b, PE/Cyanine 7, M1/70, mouse, eBioscience, 25-0112-82
anti-CD11c, PE/Cyanine 7, N418, mouse, eBioscience, 25-0114-82
anti-B220, PE/Cyanine 7, RA3-6B2, mouse, eBioscience, 25-0452-82
anti-EpCAM1, PerCP-eFluor 710, G8.8, mouse, eBioscience, 46-5891-82
anti-EpCAM1, Brilliant Violet 711, G8.8, mouse, eBioscience, 407-5791-82
UEA-1, Biotin, Vector Labs, B-1065-2
Streptavidin, PE-Cyanine7, eBioscience, 25-4317-82
anti-Ly51, PerCP-eFluor 710, BP-1, mouse, eBioscience, 46-5891-82
anti-MHCII, Alexa Fluor 700, M5/114.15.2, mouse, eBioscience, 56-5321-82
anti-CD80, Brilliant Violet 605, 16-10A1, mouse, BioLegend, 104729
anti-CD31, PE-Cyanine7, 390, mouse, eBioscience, 25-0311-82
anti-Sca-1, PE, D7, mouse, eBioscience, 12-5981-82
anti-Sca-1, FITC, D7, mouse, eBioscience, 11-5981-82
anti-Integrin7, 334908, mouse, R&D Systems, FAB3518N
Zombie Aqua Fixable Viability Kit, BioLegend, 423101

Validation

Antibodies were used at the indicated dilutions. With one exception, all antibodies are available from commercial sources, and relevant manufacturer websites contain data for reagent validation. One antibody was gifted - clone ERTR5 to detect medullary thymic epithelial cells, the original source being Dr Willem van Ewijk, Department of Cell Biology and Genetics, Erasmus University, Rotterdam (Van Vliet et al Eur J Immunol 1984 14:524). This antibody was used as neat tissue culture supernatant obtained directly from the ERTR5 hybrioma.

Animals and other research organisms

Policy information about studies involving animals; ARRIVE guidelines recommended for reporting animal research, and Sex and Gender in Research

Laboratory animals

Mus Musculus. Mice were used on either a BALB/c or B6 background as indicated. Mice were used at age 8-10 weeks and housed under barrier conditions in ventilated cage racks, as indicated in the manuscript.

Wild animals

No wild animals were used in this study.

Reporting on sex

Female mice were used in this study to avoid known differences in the thymus size of male and female mice.

Field-collected samples

No field collected animals were used in this study.

Ethics oversight

All experiments were performed at the Biomedical Services Unit at University of Birmingham, following approval by the local Animal Welfare and Ethical Review Body (AWERB) and UK national Home Office.

Note that full information on the approval of the study protocol must also be provided in the manuscript.

Flow Cytometry

Plots

Confirm that:

- [x] The axis labels state the marker and fluorochrome used (e.g. CD4-FITC).
- [x] The axis scales are clearly visible. Include numbers along axes only for bottom left plot of group (a 'group' is an analysis of identical markers).
- [x] All plots are contour plots with outliers or pseudocolor plots.
- [x] A numerical value for number of cells or percentage (with statistics) is provided.

Methodology

Sample preparation	All samples were from mouse tissues prepared using mechanical disruption or enzymatic digestion.
Instrument	Becton Dickinson Fortessa Flow Cytometer
Software	FACS DIVA software (BD Biosciences) and FlowJo software (TreeStar)
Cell population abundance	Flow cytometry cell sorting was not used in this study
Gating strategy	Initial gating was based on features of live cells using forward and side scatter. Gating for positive and negative staining was performed via omission of a primary antibody, or an isotype control.

- [x] Tick this box to confirm that a figure exemplifying the gating strategy is provided in the Supplementary Information.
